# Cannabis and cannabinoids in dermatology: a systematic review and meta-analysis of quantitative outcomes

**DOI:** 10.3389/fphar.2025.1609667

**Published:** 2025-10-17

**Authors:** Pim Sermsaksasithorn, Tanawin Nopsopon, Chatpol Samuthpongtorn, Korn Chotirosniramit, Krit Pongpirul

**Affiliations:** ^1^ Center of Excellence in Preventive and Integrative Medicine and Department of Preventive and Social Medicine, Chulalongkorn University Faculty of Medicine, Bangkok, Thailand; ^2^ School of Global Health, Chulalongkorn University Faculty of Medicine, Bangkok, Thailand; ^3^ Division of Allergy and Clinical Immunology, Department of Medicine, Brigham and Women’s Hospital, Boston, MA, United States; ^4^ Department of International Health, Johns Hopkins Bloomberg School of Public Health, Baltimore, MD, United States; ^5^ Department of Infection Biology & Microbiomes, Faculty of Health and Life Sciences, University of Liverpool, Liverpool, United Kingdom; ^6^ Clinical Research Center, Bumrungrad International Hospital, Bangkok, Thailand

**Keywords:** cannabinoids, *Cannabis sativa*, pruritus, systematic review, meta-analysis, atopic dermatitis, topical therapy

## Abstract

**Objective:**

Cannabinoids, present in *Cannabis sativa*, modulate the signaling and receptor expression of the endocannabinoid system (ECS), potentially impacting various skin conditions. However, current evaluations of their clinical effectiveness remain largely descriptive. This study aimed to quantitatively assess the effectiveness of cannabinoids, including *Cannabis sativa* extracts, in treating dermatologic disorders.

**Design:**

Systematic review and meta-analysis.

**Data sources:**

PubMed, Embase, Scopus, Web of Science, and CENTRAL from inception to 30 June 2024.

**Eligibility criteria for selecting studies:**

We included randomized controlled trials and observational studies that evaluated the efficacy or effectiveness of medical cannabis or cannabinoid-based interventions in managing dermatologic conditions, regardless of the comparator type.

**Data extraction and synthesis:**

Two reviewers independently extracted data and assessed risk of bias. Outcomes were categorized into subjective outcomes, objective outcomes, and disease-specific composite scores. Meta-analyses used standard mean differences (SMDs) with 95% confidence intervals (CIs). Study quality was assessed using RoB 2 and ROBINS-I tools.

**Results:**

We included 3,359 participants from eleven randomized controlled trials, three quasi-experimental studies, and three observational studies. Interventions included topical formulations of cannabis extract ointment, cannabidiol, N-acylethanolamine (PEA), alkylamides, and HU-210. A statistically significant reduction in pruritus was observed among participants treated with cannabinoids (SMD = −0.29, 95% CI: −0.52 to −0.06, I^2^ = 0%). No significant effects were found for skin dryness (SMD = −0.22, 95% CI: −0.58 to 0.14, I^2^ = 20%), erythema (SMD = −0.33, 95% CI: −0.65 to 0.00, I^2^ = 0%), or quality of life (SMD = −0.15, 95% CI: −0.64 to 0.34, I^2^ = 58%). Disease-specific scores for atopic dermatitis (SMD = −0.19, 95% CI: −0.44 to 0.05, I^2^ = 0%) and transepidermal water loss (SMD = 0.16, 95% CI: −0.60 to 0.93, I^2^ = 68%) also showed no significant differences.

**Conclusion:**

Cannabinoids produced a modest but statistically significant reduction in pruritus, suggesting clinical relevance for symptom management. However, no significant benefits were observed for other dermatologic outcomes. Larger, standardized randomized trials are needed to clarify their therapeutic potential in dermatology.

**Systematic Review Registration:**

identifier CRD42023397189.

## Introduction

Cannabinoids, compounds derived from *Cannabis sativa*, play a crucial role in regulating endocannabinoid system (ECS) signaling and receptor activity. The ECS is essential for maintaining the proper function of various human organs (Lowe et al., 2021; Pacher et al., 2006; Joshi and Onaivi, 2019; Meah et al., 2022; Grotenhermen, 2003), leading to a surge in research exploring the therapeutic potential of cannabis and its derivatives, particularly as their medical use becomes increasingly legalized in many countries, including Canada, Germany, Israel, and the Netherlands (Wilkinson et al., 2016; Schlag, 2020; Di Marzo et al., 2004; Pagano et al., 2022; Abuhasira et al., 2018). Several cannabis-derived extracts and synthetic cannabinoids have received FDA approval, such as Epidiolex for managing seizures, Marinol for alleviating chemotherapy-induced nausea, and Syndros for treating AIDS-related anorexia.

The skin is connected to the ECS through various mechanisms (Rio et al., 2018; Bíró et al., 2009), and ECS receptors have been identified within the skin (Baswan et al., 2020). However, cannabinoids have not yet received widespread official approval for treating skin diseases. Preclinical studies suggest they may support epidermal barrier homeostasis and reduce inflammatory responses (Gaffal et al., 2014; Kim et al., 2015). These effects are mediated through cytokine modulation, such as IL-4 and IL-13 in Th2-type allergic responses associated with atopic dermatitis (Nam et al., 2016; Abramo et al., 2017; Todurga Seven et al., 2022), and TNF-α-induced NF-κB activation and IL-17 production by Th17 cells in psoriasis (Sangiovanni et al., 2019; Guillot et al., 2014; Li et al., 2022).

Despite promising preclinical findings, consensus on the clinical use of cannabinoids in dermatology remains limited. Existing reviews are mostly qualitative, and there is still a noticeable lack of information in the thorough examination of the effectiveness of cannabinoids in dermatological uses (Shao et al., 2021; Sheriff et al., 2020; Gupta and Talukder, 2021; Eagleston et al., 2018; Milando and Friedman, 2019; Sivesind et al., 2022). Traditional assessments often rely on subjective evaluations, but the development of standardized tools—such as the Eczema Area and Severity Index (EASI), the SCORing Atopic Dermatitis (SCORAD), the Psoriasis Area and Severity Index (PASI), the visual analog scale (VAS), numerical rating scale (NRS), and verbal rating scale (VRS), and imaging technologies—now allows for more objective, quantitative analysis (Fischer et al., 1999; Jolivot et al., 2013; Chu et al., 1960; Peipert et al., 1997).

Thus, this systematic review and meta-analysis investigate whether cannabinoid-based treatments, compared to placebo or standard care, improve clinical and patient-reported outcomes in dermatological conditions and aims to quantify their efficacy while identifying gaps to inform future research.

## Methods

We conducted a systematic review and meta-analysis following the Preferred Reporting Items for Systematic Review and Meta-Analysis Protocols (PRISMA-P) statement recommendations (Shamseer et al., 2015). The review was prospectively registered with PROSPERO (CRD42023397189) and updated from a previously published protocol to include more recent data (Sermsaksasithorn et al., 2023).

### Eligibility criteria

We searched randomized controlled trials and observational studies—including cross-sectional, case–control, or cohort studies—that measured the efficacy or effectiveness of medical cannabis or cannabinoids in mitigating dermatological conditions or diseases, regardless of comparator. We excluded (1) *in vitro* studies, case reports, protocols, reviews, guidelines, editorials, commentaries, and letters; (2) non-peer-reviewed publications; (3) animal studies; (4) articles not published in English. All identified studies were imported into Covidence for de-duplication. Paired reviewers independently evaluated titles and abstracts. Abstracts not reporting the therapeutic effects of cannabis and cannabinoids on dermatological conditions or diseases were omitted. Those retained proceeded to a full-text review, adhering to all eligibility criteria, with reasons for exclusion documented. Discrepancies were resolved through discussion or adjudication when necessary.

### Clinical outcomes

Outcomes focused on improvements in dermatological characteristics were systematically categorized into

1. Generic Outcomes:
  a. Subjective—either clinical or patient-reported outcomes.
  b. Objective outcomes, assessed by standardized instruments.
2. Disease-Specific Composite Scores, entailing a mix of subjective and objective outcomes, utilizing indices like the Psoriasis Area Severity Index (PASI), the Eczema Area and Severity Index (EASI), and the SCORing Atopic Dermatitis (SCORAD) tools

When studies reported multiple time points, we extracted data from the longest follow-up.

### Search strategy

We searched through five databases: PubMed, Embase, Scopus, Web of Science, and CENTRAL from inception to 30 June 2024. The terms for the search strategy are presented in Supplementary Appendix 1.

### Data extraction

Two reviewers separately extracted data, including (1) study identifiers such as authors, publication year, design, journal, contact details, country, and funding source; (2) participant demographics and clinical characteristics; (3) details of cannabinoid interventions (formulation, route, and duration); (4) control preparation; (5) results and time points; and (6) interpretative discussions. Corresponding authors were contacted to obtain any partially reported data. If no response was received within 14 days, the analyses proceeded using the available data.

### Quality assessment

The two reviewers independently gauged the risk of bias of each study. For all randomized controlled studies, we employed the Cochrane Risk-of-Bias 2 (RoB2) tool, evaluating various domains including the randomization process, allocation concealment, participant and outcome evaluator blinding, adequacy of outcome data, selective outcome reporting, and other potential bias sources. For non-randomized studies, we utilized the ROBINS-1 tool, assessing confounding, selection, intervention classification, deviations from intended interventions, missing data, outcome measurement, and reported result selection. Discrepancies between the two reviewers were resolved by discussion or, if unresolved, by an adjudicator. The adjudicator had no conflicts of interest or affiliations with the research group.

### Data synthesis and analysis of heterogeneity

Effect sizes and their respective 95% confidence intervals (CIs) were pooled using random-effects models. For continuous outcomes in controlled studies, we employed the standard mean difference (SMD) between pre- and post-performance in cases and controls, mitigating baseline heterogeneity between them. A *p*-value threshold of 0.05 was deemed statistically significant. Heterogeneity was quantified using Cochran’s Q-test (*p*-value of 0.10 indicating heterogeneity) and the Higgins’ test (*I* ^*2*^) (with <25% defined as low, 25%–75% as moderate, and >75% as high heterogeneity) (Higgins and Thompson, 2002). Sensitivity analyses were enacted by iteratively excluding one study at a time to ascertain the statistical resilience of the primary outcome. Publication bias was evaluated through the Egger’s regression asymmetry test and funnel plots in R version 4.0.1. Egger’s test was omitted when only two studies were available for an outcome. All meta-analyses were conducted using RevMan 5.4 (The Cochrane Collaboration, The Nordic Cochrane Centre, Copenhagen, Denmark).

### Patient and public involvement

There was no direct patient participation in formulating the research question, determining outcome measures, or designing and conducting the study.

### Risk of bias

The overall risk of bias in the 17 included studies was unclear to high, as shown in Table 1.

**TABLE 1:** The overall risk of bias for the studies included in the meta-analysis, including the RoB 2 domains (Table 1A) and the ROBINS-I domains (Table 1B).

A						
Study- RoB2 domain	Randomization	Deviation from intended protocol	Missing data	Measurement of outcome	Selection of result	Overall risk of bias
Callaway et al. (2005)	Low	Some concerns	High	Low	Some concerns	High
Dvorak et al. (2003)	Low	Low	Low	Low	High	High
Gao et al. (2022)	Low	Low	Some concerns	Low	Low	Some concerns
Grant et al. (2022)	Low	Low	Low	Some concerns	High	High
Oláh et al. (2017)	Low	Low	High	Low	High	High
Rao et al. (2023)	Low	Low	Low	Some concerns	High	High
Rukwied et al. (2003)	Some concerns	Some concerns	Low	Some concerns	Some concerns	High
Spiera et al. (2023)	Low	Low	Low	Some concerns	Low	Some concerns
Visse et al. (2017)	Low	High	High	High	High	High
Werth et al. (2022)	Low	Low	Low	Low	High	High
Yuan et al. (2014)	Low	Low	Some concerns	Low	High	High

B								
Study- ROBINS-1 domain	Confounding	Selection of participants into study	Classification of interventions	Deviations from intended interventions	Missing data	Measurement of outcome	Selection of result	Overall risk of bias
Ali et al. (2015)	Low	Some concerns	Some concerns	Low	Low	High	Low	High
Ali et al. (2020)	Low	Some concerns	Low	Low	Low	Some concerns	Low	Some concerns
Eberlein et al. (2008)	High	Low	Low	Some concerns	Some concerns	High	Low	High
Palmieri et al. (2019)	Low	Low	Low	Some concerns	Some concerns	High	High	High
Szepietowski et al. (2005)	Low	Low	Low	Some concerns	Some concerns	High	Low	High
Vitek et al. (2024)	Low	High	Low	Some concerns	Some concerns	High	High	High

## Results

### Study selection

**FIGURE 1:**
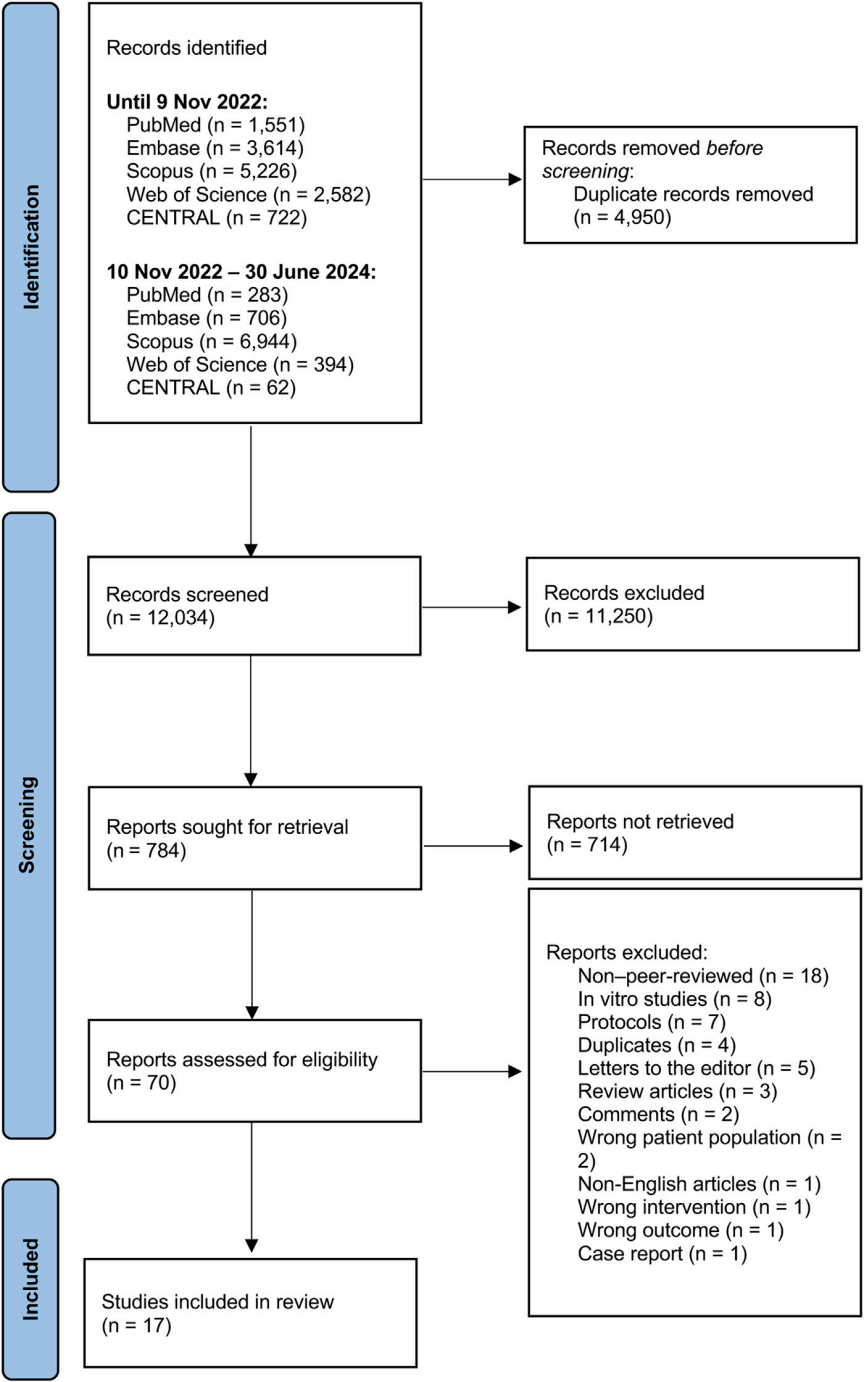
PRISMA flow diagram.

A total of 12,034 studies were initially screened. Seventy full-text studies were evaluated for eligibility, with 53 being excluded due to the following reasons: eighteen were non-peer-reviewed (conference abstracts), eight were *in vitro* studies, seven were protocols, four were duplicates, five were letters to editor, three were review articles, two involved incorrect population, two were commentaries, and one each was a case report, non-English study, incorrect intervention, or had irrelevant outcomes. Seventeen studies were incorporated into the meta-analysis (Figure 1).

### Study characteristic

Study characteristics are summarized in the Supplementary Table 1. The final set included eleven randomized controlled trials, three quasi-experimental studies, and three observational studies, engaging 3,359 participants aged 2–80 years. Supplementary Table 2 presents a comparison of the studies included in the meta-analysis based on key intervention characteristics, including cannabinoid type, dosage, and formulation.

#### Subjective outcomes

##### Pruritus

Eight studies scrutinized the pruritus score, six with control groups and two without (Eberlein et al., 2008; Visse et al., 2017; Szepietowski et al., 2005; Dvorak et al., 2003; Callaway et al., 2005; Yuan et al., 2014; Rao et al., 2023; Werth et al., 2022). Among the studies with control groups, variances in measurement scales were noted: two used a VAS, another applied both a VAS and a 1–5-point VRS, one incorporated a 0–5-point VRS, one used a 0–100-point NRS, and the remaining study used a 0–9-point scale NRS.

**FIGURE 2:**
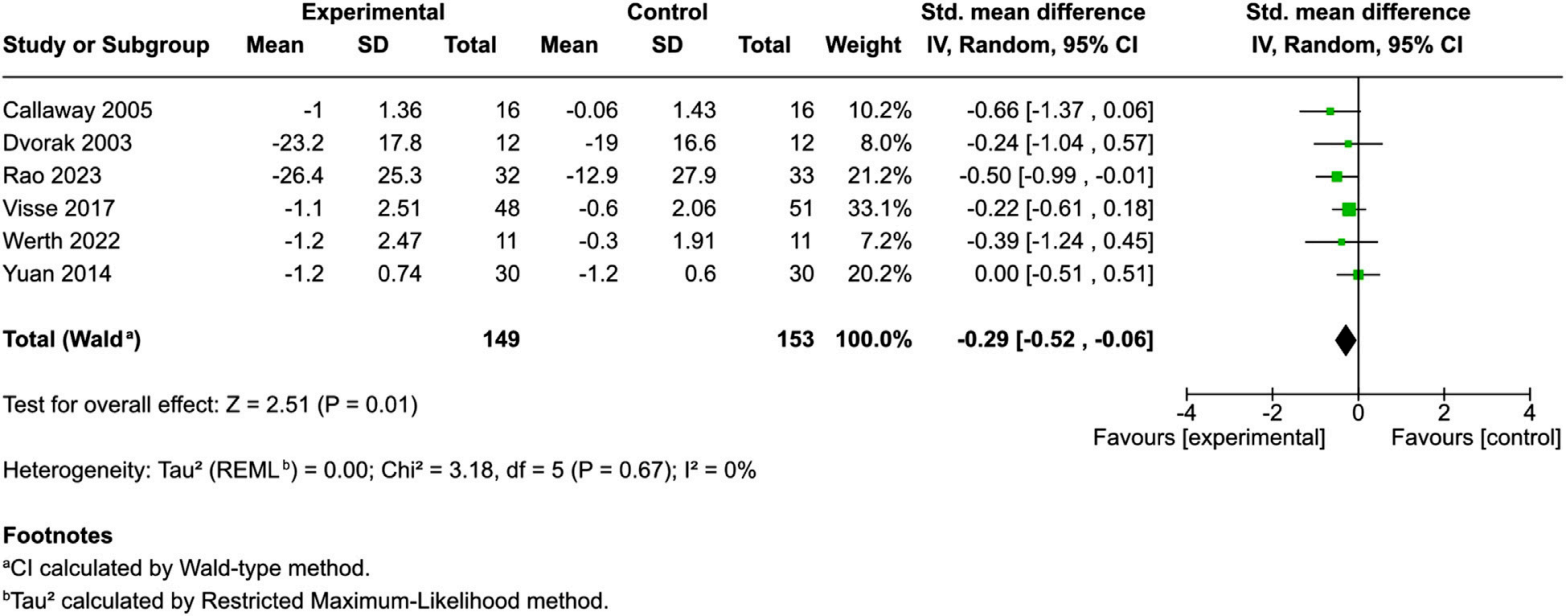
Forest plot of pruritus scores between participants receiving cannabinoids or cannabis and controls.

Patients were administered different cannabinoid or cannabis products [hempseed oil (Callaway et al., 2005), HU-120 skin patch (a synthetic CB-1 and CB2 agonist) (Dvorak et al., 2003), N-palmitoylethanolamine cream (Visse et al., 2017; Rao et al., 2023), N-palmitoylethanolamine and N-acetylethanolamine cream (Yuan et al., 2014), or lenabasum (Werth et al., 2022)]. Meta-analysis showed a small but significant reduction in pruritus scores between the cannabinoid/cannabis and control recipients [SMD = −0.29, 95% CI: −0.52 to −0.06, I^2^ = 0%] (Figure 2). Controls included ethanol (Dvorak et al., 2003), body lotion (Visse et al., 2017), olive oil (Callaway et al., 2005), and emollients (Yuan et al., 2014; Rao et al., 2023). A subgroup analysis of products containing palmitoylethanolamine (Visse et al., 2017; Yuan et al., 2014; Rao et al., 2023) found no significant effect on pruritus scores (SMD = −0.24, 95% CI: −0.5 to 0.02, I^2^ = 0%). Two uncontrolled studies used a VAS and showed potential benefit but emphasized the need for further controlled trials (Eberlein et al., 2008; Szepietowski et al., 2005).

##### Skin dryness

Six studies evaluated skin dryness (Eberlein et al., 2008; Szepietowski et al., 2005; Callaway et al., 2005; Yuan et al., 2014; Rao et al., 2023; Oláh et al., 2017), with four using control groups. Three provided mean and standard deviation data. One additional controlled study did not offer data regarding mean and standard deviation but reported a significant reduction in dryness at day 85 (p = 0.028) (Oláh et al., 2017).

**FIGURE 3:**
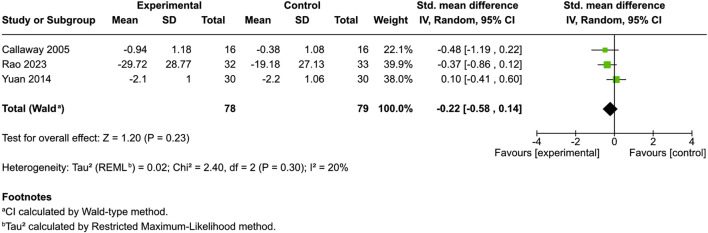
Forest plot of skin dryness between participants receiving cannabinoids or cannabis and controls.

An evaluation of patients each in the different cannabinoid [hempseed oil (Callaway et al., 2005), N-palmitoylethanolamine cream (Rao et al., 2023) and emollient PEA/AEA cream (Yuan et al., 2014)] and control cohorts revealed no significant divergence in skin dryness between the groups (SMD = −0.22, 95% CI: −0.58 to 0.14, I^*2*^ = 20%) (Figure 3). The two uncontrolled studies reported improvements, but results remain inconclusive without comparators (Eberlein et al., 2008; Szepietowski et al., 2005).

##### Erythema

**FIGURE 4:**
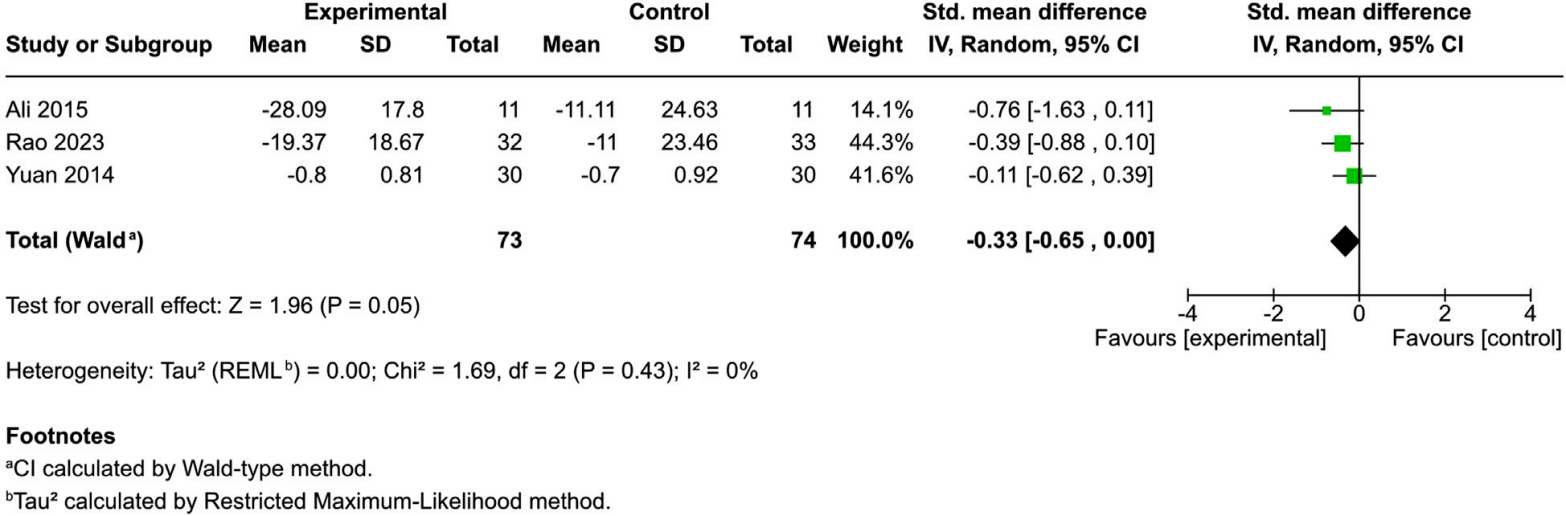
Forest plot of skin erythema between participants receiving cannabinoids or cannabis and controls.

Four controlled studies addressed erythema; three provided mean and SD values: one used a 0–4 score (Ali and Akhtar, 2015), another used a 0–100 score (Rao et al., 2023), and a third used a 0–9 dermatologist-assessed scale (Yuan et al., 2014). The remaining study simply indicated a significant enhancement in physician-assessed erythema among cannabis users compared to a reference product (Oláh et al., 2017; Vitek and Matjaž, 2024). Among patients administered distinct cannabinoids [3% *Cannabis sativa* extract (Ali et al., 2020), N-palmitoylethanolamine cream (Rao et al., 2023), and emollient PEA/AEA cream (Yuan et al., 2014)], juxtaposed with control patients, no significant difference in skin erythema was observed (SMD = −0.33, 95% CI: −0.65 to 0.00, I^2^ = 0%) (Figure 4). However, in Vitek’s study, an objective erythema index measured with a Mexameter demonstrated decreased erythema from baseline, with the greatest change discovered in the hempseed oil group (Vitek and Matjaž, 2024).

##### Scaliness and roughness

In a controlled trial examining patient-evaluated skin roughness and scaliness (Visse et al., 2017), no discernible difference was identified between lotions with and without palmitoylethanolamide (PEA). In a pre- and post-intervention study, improvements in scaling, lichenification, and excoriation were observed in 71.6% of patients at baseline, increasing to 82.5% following treatment.

##### Nociceptive properties

Rukwied’s study used capsaicin-induced nociception to evaluate pressure sensitivity, heat pain threshold, pinprick hyperalgesia, and touch allodynia. A reduction was observed solely in the intensity of heat pain upon cannabinoid usage (*p* = 0.01), while no notable variations were identified across other domains (Rukwied et al., 2003). Additionally, Yuan’s study did not demonstrate any alteration in sensory threshold by the 5 Hz cold pressor test (CPT) (Yuan et al., 2014).

##### Quality of life

**FIGURE 5:**
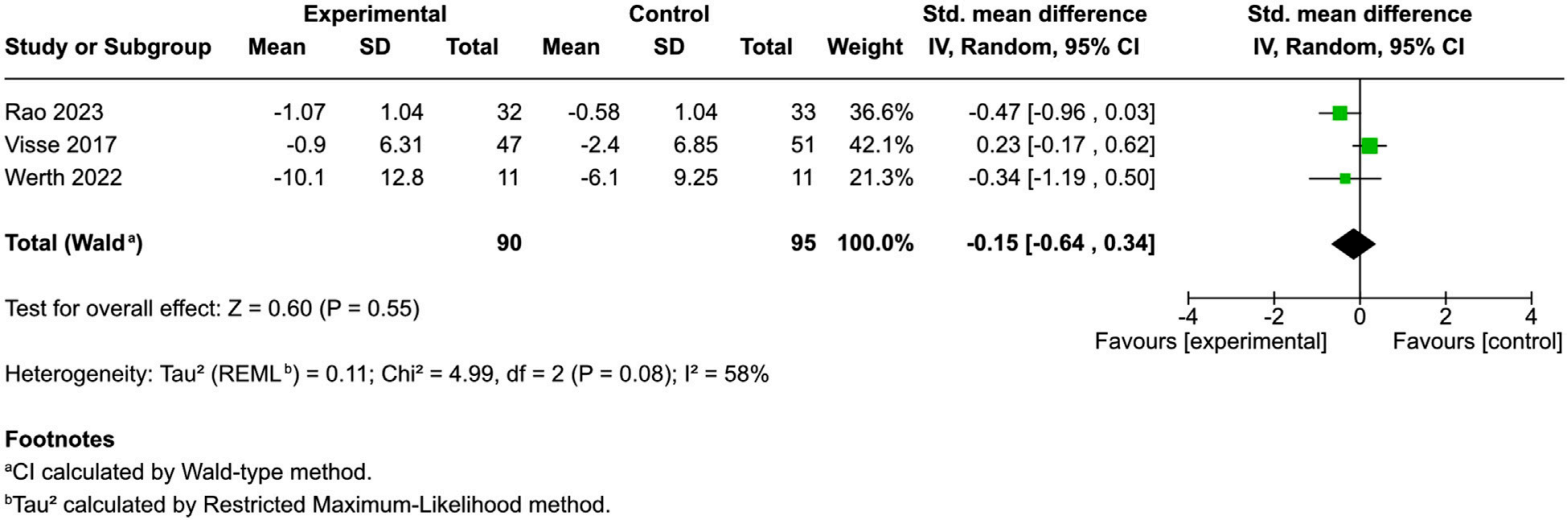
Forest plot of quality of life between participants receiving cannabinoids or cannabis and controls.

Two studies using the Dermatology Life Quality Index (DLQI) for atopic dermatitis and dry skin and one study using Skindex-29+3 for dermatomyositis (Werth et al., 2022; Visse et al., 2017; Rao et al., 2023) found no significant disparity between the intervention and control (SMD = −0.15, 95% CI: −0.64 to 0.34, I^*2*^ = 58%) (Figure 5). Conversely, Eberlein’s research revealed substantial improvement in 56.3% of cases, with 83% of patients noting improved global assessment and better sleep quality (Eberlein et al., 2008). Szepietowski’s study indicated that 90.5% of patients with uremic pruritus experienced satisfaction following product application, whereas sleep disturbances markedly increased upon discontinuation (Szepietowski et al., 2005).

#### Disease-specific composite score (mixed subjective and objective outcome)

##### Atopic dermatitis severity score

**FIGURE 6:**
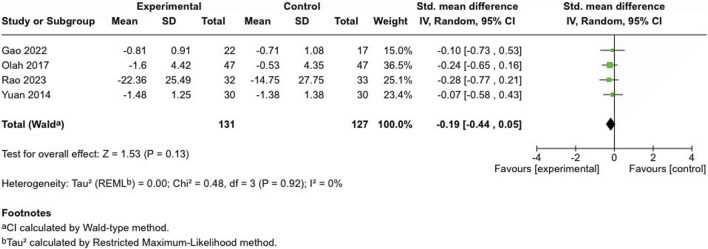
Forest plot of atopic dermatitis severity scores between participants receiving cannabinoids or cannabis and controls.

Four controlled studies assessed atopic dermatitis severity: two used the Eczema Area Severity Index (EASI) (Yuan et al., 2014; Rao et al., 2023), one employed the Investigator’s Global Assessment (IGA) score, ranging from 0 to 4 (Gao et al., 2022), and another utilized the SCORing Atopic Dermatitis (SCORAD) method (Oláh et al., 2017). No significant difference was noted between cannabinoid-treated and control groups (SMD = −0.19, 95% CI: −0.44 to 0.05, I^*2*^ = 0%) (Figure 6).

##### Psoriasis severity score

A study by Palmieri, which used the *Psoriasis* Area Severity Index (*PASI*) score, identified significant improvement by day 90 (p < 0.001) (Palmieri et al., 2019).

##### Diffuse cutaneous systemic sclerosis severity score

No significant difference was found in the American College of Rheumatology combined response index in diffuse cutaneous systemic sclerosis (dcSSc) or the modified Rodnan skin thickness score (MRSS) at week 52 between patients using lenabasum 20 mg twice a day and those taking a placebo (Spiera et al., 2023).

##### Dermatomyositis severity score

In the lenabasum-treated group (20 mg daily for 28 days, then twice daily for 56 days), the Cutaneous Dermatomyositis Disease Area and Severity Index (CDASI) became significantly improved 4 weeks after discontinuation (day 113), suggesting a continued anti-inflammatory effect post-treatment (Werth et al., 2022).

#### Objective outcomes

##### Skin barrier function

Trans epidermal water loss (TEWL) was evaluated in four studies—two controlled (Callaway et al., 2005; Yuan et al., 2014) and two uncontrolled (Vitek and Matjaž, 2024; Palmieri et al., 2019). All demonstrated a significant reduction from baseline (p < 0.05).

**FIGURE 7:**
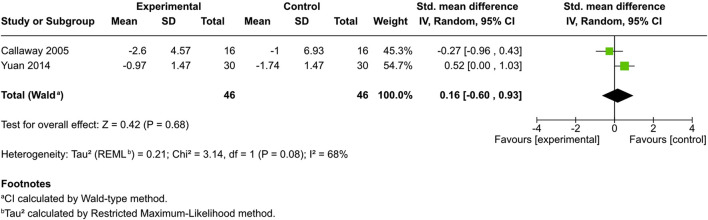
Forest plot of skin barrier function evaluations between participants receiving cannabinoids or cannabis and controls.

Among the controlled studies, no significant difference was identified in the cannabinoid-treated and control groups (SMD = 0.16, 95% CI: −0.6 to 0.93, I^2^ = 68%) (Figure 7).

##### Skin hydration level

Three studies investigated skin hydration (Yuan et al., 2014; Vitek and Matjaž, 2024; Palmieri et al., 2019). Yuan’s study highlighted a significantly greater increase in skin surface capacitance with PEA/AEA cream than the control (p < 0.05). Palmieri’s study and Vitek’s study demonstrated an enhancement in skin hydration following treatment, albeit without control groups.

##### Skin surface properties

Ali’s study (Ali et al., 2020) solely explored skin topography assessments using Visioscan^®^ VC 98. Skin texture parameters, such as energy, contrast, and variance, revealed significant improvements post-treatment. The research also indicates that cannabis seed extract significantly impacted SEr (skin roughness), SEsc (skin scaliness), SEsm (skin smoothness), and SEw (skin wrinkles) across all reading intervals, a result not mirrored by the base lotion.

##### Skin blood flow and axonal reflex flare

Dvorak’s study (Dvorak et al., 2003) demonstrated a notable reduction in skin blood flow and axonal reflex flare following HU210, compared to a control group (p < 0.05 and p < 0.03, respectively).

##### Skin elasticity

Palmieri’s study (Palmieri et al., 2019) was the only one to assess elasticity, reporting a significant improvement from baseline to end of study (p < 0.001) (Eberlein et al., 2008).

##### Sebum level

In a study utilizing a sebumeter to measure cheek skin sebum (Ali and Akhtar, 2015), the side treated with a base plus 3% cannabis seed extract exhibited a significant decrease (p < 0.05) compared to the side treated with the base alone.

##### Lipid lamellae analysis

Oláh et al. (2017) observed increased levels of overall epidermal lipids, ceramide EOS, cholesterol, and numbers of intercellular lipid lamellae in the intercellular space, assessed through transmission electron microscopy.

##### Melanin content

In Vitek’s study, melanin content measured by a Mexameter was not affected by lyotropic liquid crystal products containing hempseed oil (Vitek and Matjaž, 2024).

##### Publication bias

Funnel plots illustrated the dispersion and heterogeneity of the data for each outcome. The *I* ^*2*^ value indicated no heterogeneity for pruritus, erythema, and atopic dermatitis severity scores; low heterogeneity for skin dryness; and moderate heterogeneity for quality of life and transepidermal water loss. The Egger’s regression asymmetry showed no significant publication bias for the pruritus score (p = 0.49), skin dryness (p = 0.69), skin erythema (p = 0.44), quality of life (p = 0.61), and atopic dermatitis severity scores (p = 0.85). Supplementary Figures 1–5 present the respective funnel plots.

## Discussion

Our review stands as the initial meta-analysis evaluating the therapeutic potency of cannabis and cannabinoids in dermatological outcomes. We incorporated 17 studies covering a spectrum of conditions, encompassing atopic dermatitis, psoriasis, atopic eczema, dry and itchy skin, visible flexural dermatitis, acne vulgaris, diffuse cutaneous systemic sclerosis (dcSSc), dermatomyositis, chronic pruritus, uremic pruritus, and experimental dermatological scenarios, including capsaicin-induced pain, hyperalgesia, histamine iontophoresis, and dialysis. This analysis offers insights into cannabinoids’ potential for various dermatological issues.

This meta-analysis suggests that cannabinoids and cannabis-based interventions may offer a modest but statistically significant improvement in pruritus symptoms compared to control treatments (SMD = −0.29, 95% CI: −0.52 to −0.06, I^2^ = 0%). This finding aligns with the proposed antipruritic effects of cannabinoids, which are thought to be mediated through peripheral CB1/CB2 receptors and modulation of cutaneous nerve endings and inflammatory pathways (Misery et al., 2021; Yoo and Lee, 2023; Ramer and Hinz, 2022; Avila et al., 2020). The consistency across studies (I^2^ = 0%) supports the reliability of this effect across different cannabinoid formulations and study designs.

No statistically significant improvements were observed in other subjective outcomes, including skin dryness (SMD = −0.22), erythema (SMD = −0.33), or quality of life (SMD = −0.15). Similarly, no significant changes were found in disease-specific severity scores for atopic dermatitis (SMD = −0.19) or in objective skin barrier measures such as transepidermal water loss (SMD = 0.16). The lack of significant effects on inflammation and skin barrier function suggests that cannabinoids may primarily target sensory symptoms like pruritus, rather than the structural or immunologic components of dermatologic disease (Dvorak et al., 2003; Ständer et al., 2005).

However, several factors may account for these limited effects. First, cannabinoids such as CBD and THC are lipophilic, making their absorption through the skin highly dependent on formulation. The lack of standardized delivery vehicles across studies likely affected bioavailability and therapeutic efficacy, especially when targeting deeper skin layers or immune cells involved in inflammatory processes (Lapteva et al., 2024; Bruni et al., 2018). Second, the duration of most studies may have been insufficient to observe meaningful clinical improvements. For instance, in the study by Oláh et al. (2017), a significant improvement in SCORAD score was detected only at day 85, with no such changes noted at days 29 or 57. In contrast, other studies included in the meta-analysis assessed atopic dermatitis severity over shorter durations, ranging from 14 days to 4 weeks, potentially underestimating the treatment effect. Third, variability in trial design—such as the use of non-standardized outcome measures and differences in concomitant treatments—introduced heterogeneity that limits the reliability of pooled results.

Overall, cannabinoids may serve as adjunctive agents for pruritus relief but are not yet supported as comprehensive treatments for inflammatory skin disorders. More robust, large-scale randomized trials—using standardized delivery methods, longer study durations, and consistent outcome measures—are needed to clarify the clinical efficacy of cannabinoid-based therapies in dermatology.

## Strengths and limitations

Existing evaluations on this subject pertaining to skin problems are scant. Our research conducted quantitative analyses through the utilization of systematic reviews and meta-analyses. This is the initial article that divides outcomes into disease-specific, subjective, and objective. This structured approach enhances clarity, reproducibility, and applicability for future research, reduces author bias, and may help identify common features across various skin diseases.

However, the review faces several methodological challenges. Most included studies were of low quality, limiting the generalizability of our findings. Due to the high or unclear risk of bias in included studies, we reviewed relevant letters, including one by Tammaro et al. (2019), which reported partial or complete healing in 50 patients with mild to moderate psoriasis, atopic dermatitis, and irritative contact dermatitis after 12 weeks of cannabis cream. However, it was excluded from our meta-analysis as it was a letter to the editor and assessed different outcomes from our primary focus. The small number of studies and participants for each outcome, along with inconsistent outcome measures—even for identical conditions—further weakens reliability. Some studies used non-standard or self-devised scoring systems, such as the pruritus scale by Callaway and Yuan et al. To address variability, we applied the standard mean difference in our meta-analysis (Murad et al., 2019), though this approach’s potential for small effect sizes emerged as a limitation (Cuijpers, 2021). Additional heterogeneity arose from differences in treatment duration, outcome time points, and product composition—especially when formulations contained other active ingredients in addition to cannabinoids. For instance, Eberlein et al. used a lotion containing multiple components, making it difficult to isolate cannabinoid effects in the absence of control groups (Eberlein et al., 2008). Potential publication bias is another concern. Although we conducted comprehensive searches and included gray literature, Egger’s test—used to detect bias—has limited sensitivity when fewer than ten studies are available (Furuya-Kanamori et al., 2020), as was the case for most outcomes. Language bias may have affected results, as we included only English-language publications or translations, possibly excluding relevant data from non-English sources where cannabinoid research is growing.

### Comparison with other studies

Previous scoping studies and systematic reviews have focused on different skin diseases, showing that cannabinoids exhibit potential as therapeutics for conditions such as contact dermatitis, atopic dermatitis, psoriasis, acne, seborrhea, skin cancer, and idiopathic pruritus (Baswan et al., 2020; Eagleston et al., 2018; Sivesind et al., 2022; Filipiuc et al., 2023; Martins et al., 2022). Unlike our study, these reviews did not concentrate on quantitative effects and specifically targeted skin diseases rather than conditions. The majority of them concur that additional randomized controlled trials and studies employing standardized treatment protocols are imperative to gain greater clarity of the effectiveness of cannabinoids (Baswan et al., 2020; Eagleston et al., 2018; Sivesind et al., 2022; Filipiuc et al., 2023; Martins et al., 2022; Popp et al., 2023).

### Policy implications and conclusions

This is the first meta-analysis to systematically quantify cannabinoid efficacy using a structured framework of disease-specific, subjective, and objective outcomes. While cannabinoids show promise for symptom relief in pruritus, current evidence does not support their broad application for inflammatory or barrier-related skin disorders. Methodological limitations—including small sample sizes, variable formulations, short treatment durations, and inconsistent outcome reporting—restrict the generalizability of findings. Given current evidence, cannabinoids should not yet be recommended as standalone treatments for dermatologic conditions. However, their antipruritic effects suggest a promising adjunctive role, particularly for patients with chronic itch unresponsive to standard therapies. To enable integration into clinical practice, robust research is essential. Policymakers should support high-quality randomized trials and develop clear guidelines for topical cannabinoid use in dermatology, especially for pruritus management.

### Unanswered questions and future research

Unanswered questions remain, as current data are insufficient to confirm the efficacy of cannabis and cannabinoids for skin conditions. To determine their clinical value, future studies should employ standardized and validated outcome measures, extend follow-up durations to assess sustained effects, ensure consistency in cannabinoid formulation and delivery with known bioavailability profiles, and address blinding challenges through careful study design. These methodological refinements will improve comparability across studies and strengthen the evidence base needed for clinical decision-making in dermatologic practice.

### Dissemination to participants and related patient and public communities

Results will be disseminated within healthcare communities using social media, international conferences, and non-profit organizations.

### Protocol access

The reviewed protocol can be accessed via https://bmjopen.bmj.com/content/13/9/e075007.

## Data Availability

The original contributions presented in the study are included in the article/Supplementary Material, further inquiries can be directed to the corresponding author.
