# Supplementary figures and images for: Cannabis and cannabinoids in dermatology: a systematic review and meta-analysis of quantitative outcomes
Source: Front Pharmacol. 2025 Oct 17;16:1609667. doi: 10.3389/fphar.2025.1609667 (PMC12575346; doi:10.3389/fphar.2025.1609667)

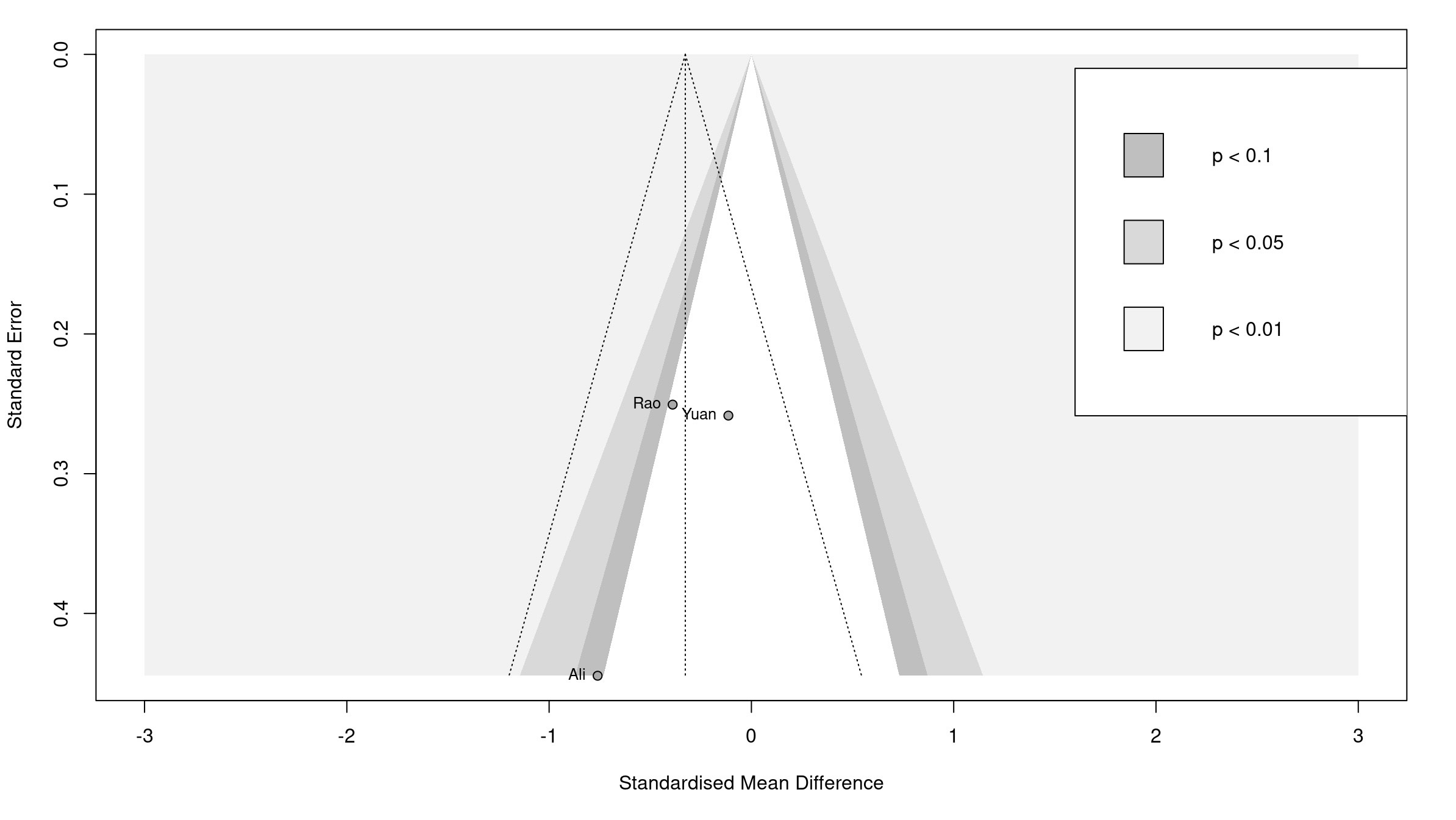

Supplement: Supplementary file 1 [file Image3.jpeg]

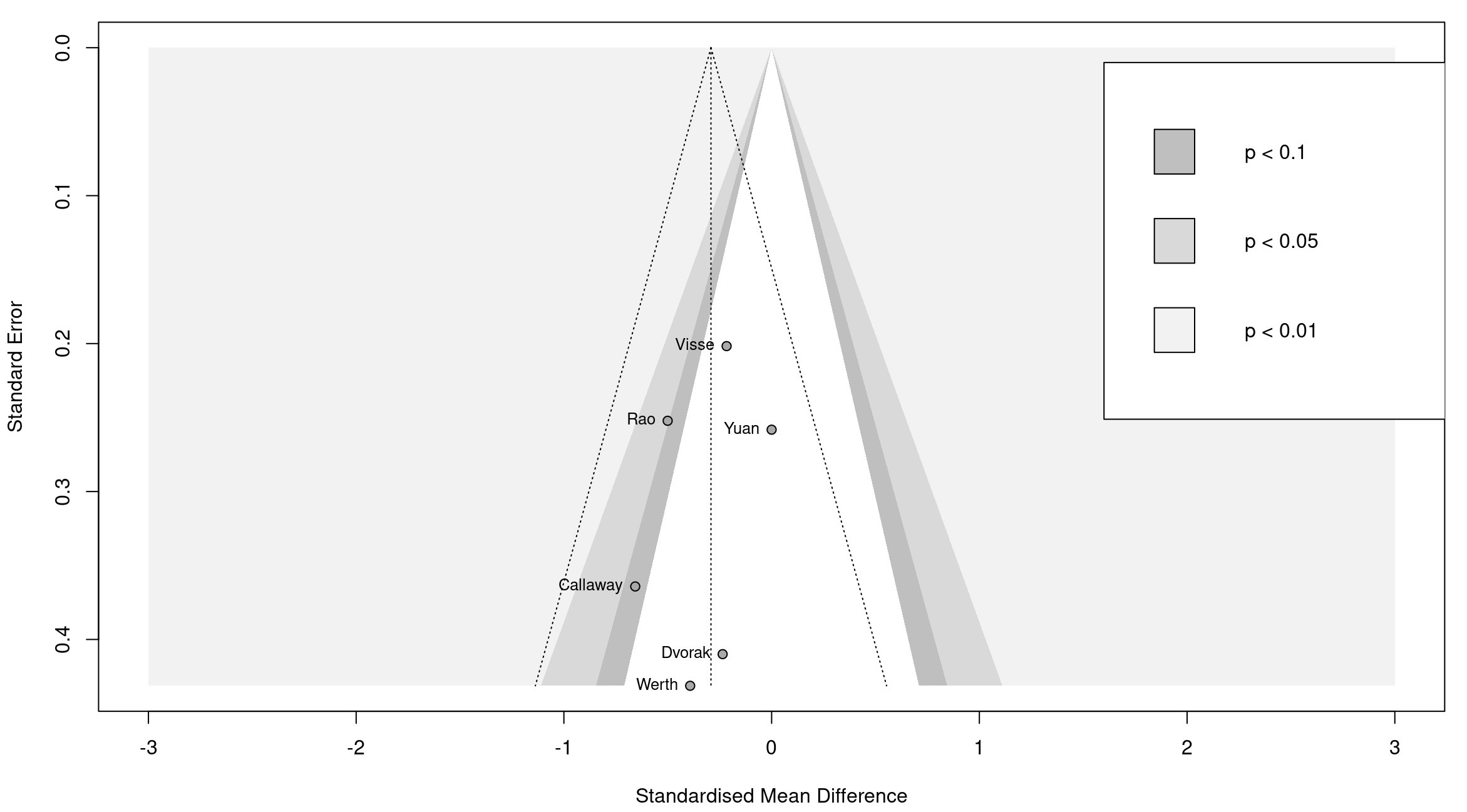

Supplement: Supplementary file 2 [file Image1.jpeg]

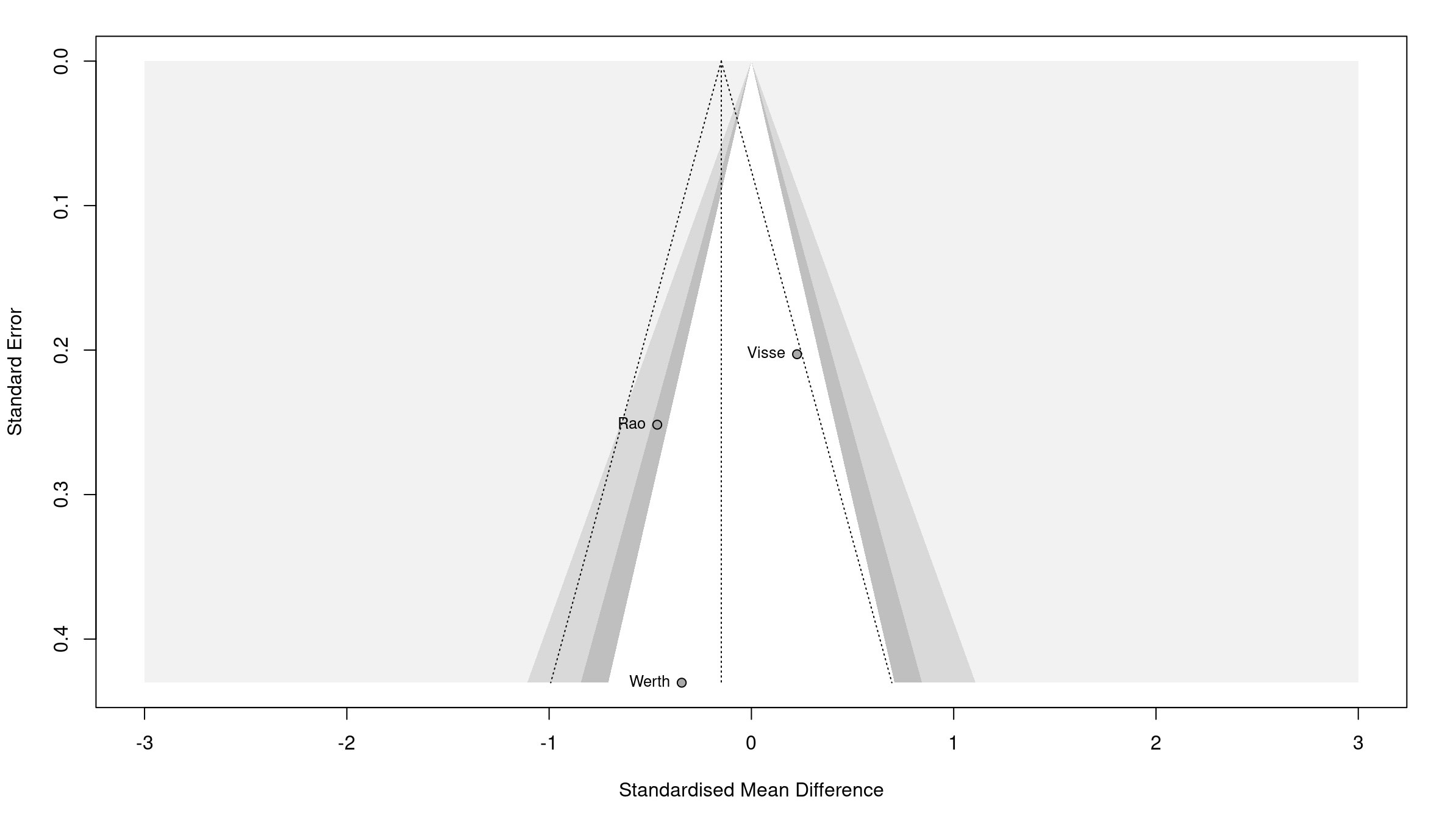

Supplement: Supplementary file 3 [file Image4.jpeg]

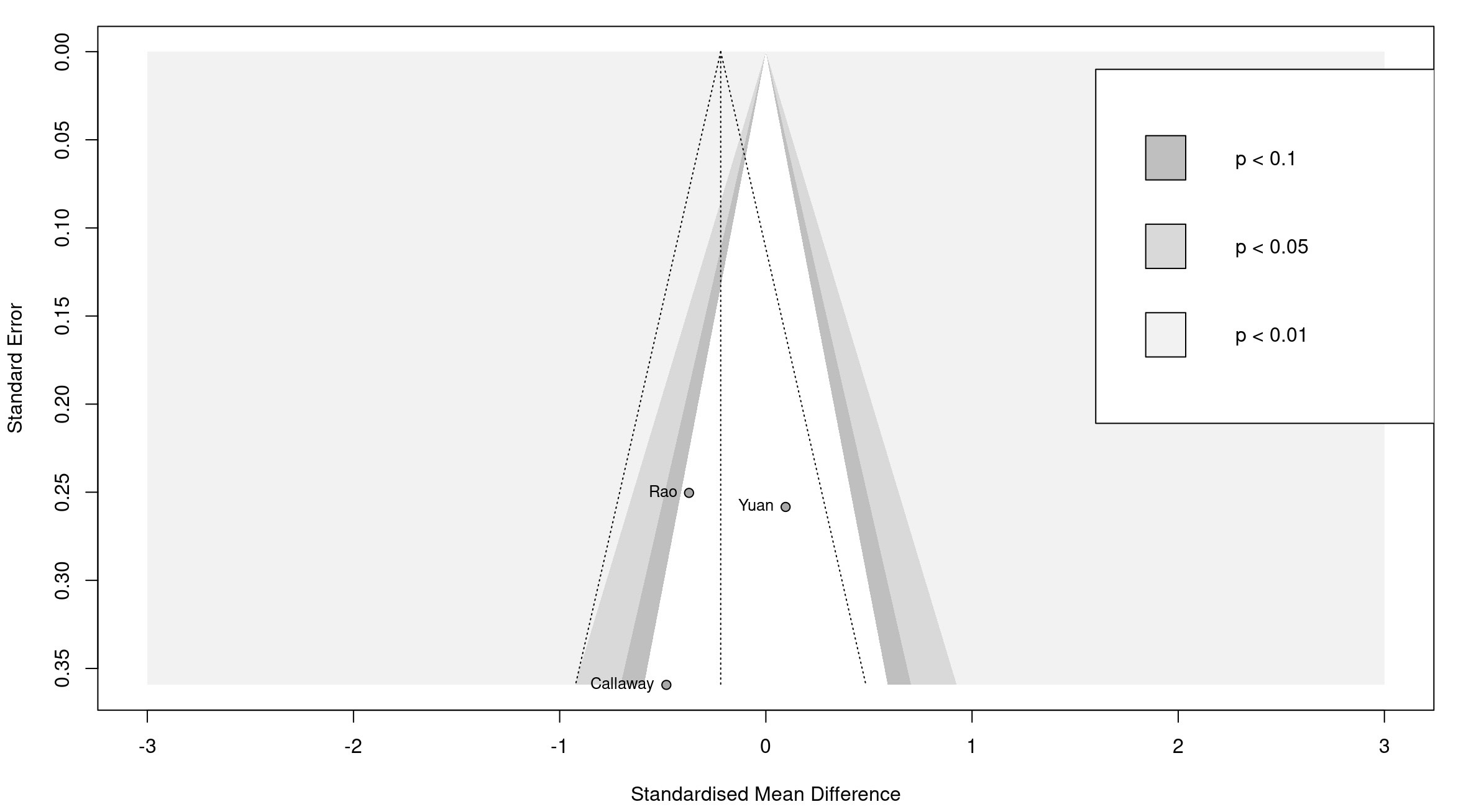

Supplement: Supplementary file 4 [file Image2.jpeg]

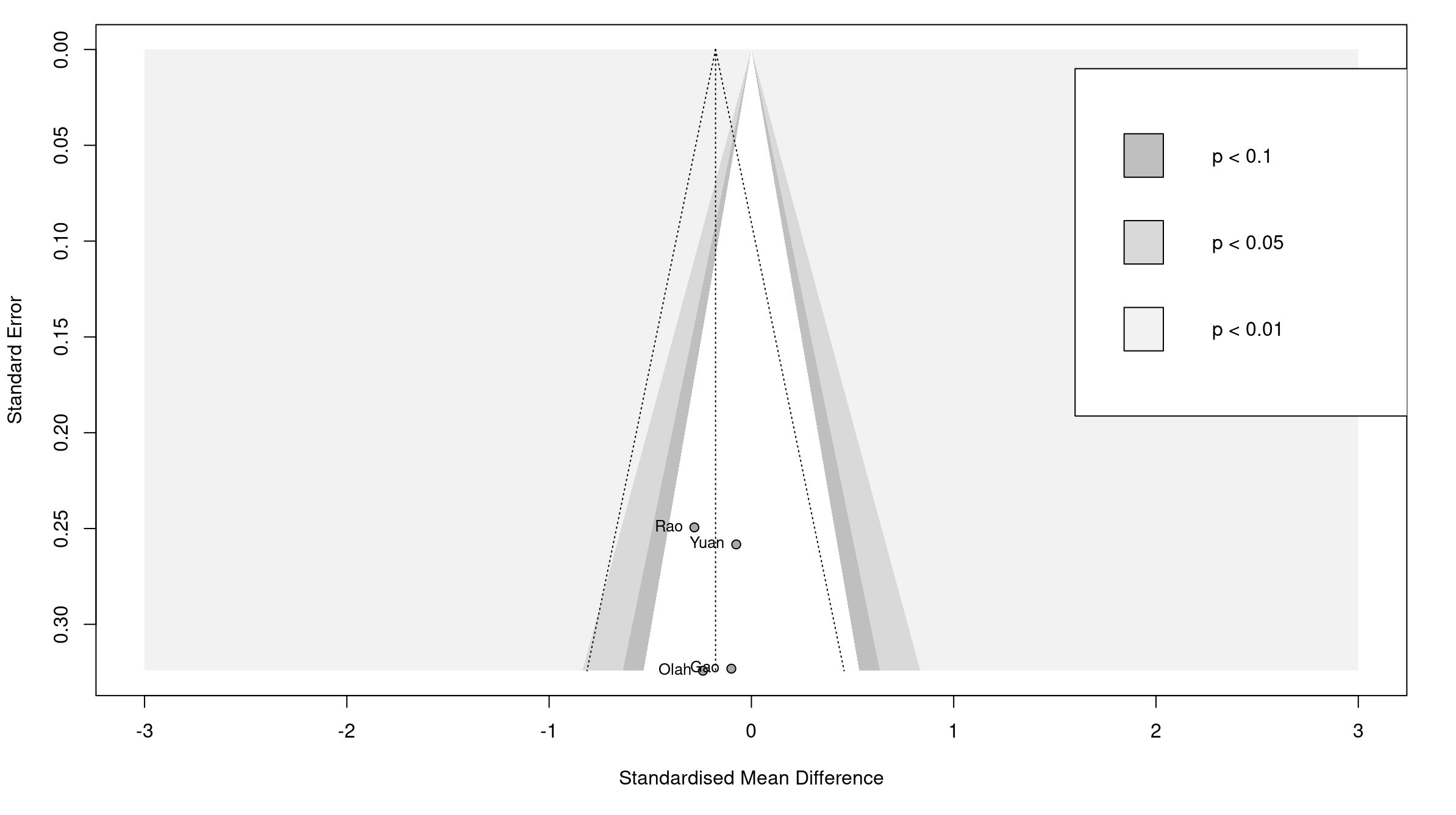

Supplement: Supplementary file 5 [file Image5.jpeg]
