# Supplementary material for: Cannabis and cannabinoids in dermatology: a systematic review and meta-analysis of quantitative outcomes
Source: Front Pharmacol. 2025 Oct 17;16:1609667. doi: 10.3389/fphar.2025.1609667 (PMC12575346; doi:10.3389/fphar.2025.1609667)
Supplement: Supplementary file 6 [file Supplementaryfile1.zip › Supplementary table 1.DOCX]

|  | **Country** | **Type of cannabinoids** | **Study Design** | **Interventions** | **Route** | **Sample**  **Size** | **Conditions** | **Outcomes** |
| --- | --- | --- | --- | --- | --- | --- | --- | --- |
| **Eberlein et al.** | Brazil Germany  Spain  Phillippine | PEA/AEA | Observational study | Physiogel A.I. Cream | Topical | 2456 | Atopic Eczema,  Dry skin,  Itchy skin,  Visible flexural dermatitis | (1) Physician’s assessment of erythema, dryness, scaling, lichenification, excoriation, itching, global assessment of improvement and flares up  (2) Patient’s assessment of pruritus VAS, intensity of itch, sleep quality, global assessment of improvement, skin care properties including absorption and distribution |
| **Visse et al.** | Germany | PEA lotion | Randomized control trial | Physiogel^®^ Body Lotion | Topical | 100 | Chronic Pruritus,  Dry skin | (1) Pruritus, VAS  (2) Pruritus, VRS  (3) Stinging sensation  (4) Skin roughness  (5) Scaling  (6) Skin tightness  (6) Chronic scratch lesions  (7) Dermatology Life Quality Index  (8) Global patient-defined treatment benefit  (9) Sleeping problems |
| **Szepietowski et al.** | Italy | PEA/AEA | Observational study | Physiogel A.I. Cream | Topical | 21 | Uremic prutirus | (1) Pruritus, VAS  (2) Pruritus score scale  (3) Xerosis score  (4) Tolerance  (5) Global agreement of patients |
| **Dvorak et al.** | United Kingdom | HU-210 | Randomized control trial | (1) HU-210 Skin patch  (2) HU-210 infused microdialysis | Patch  Micro-  dialysis | 12  6 | Healthy Volunteer with Histamine iontophoresis  Healthy Volunteer with Microdialysis | (1) Skin Blood flow  (2) Pruritus, VAS  (1) Blood flow  (2) Axon flare reflex |
| **Callaway et al.** | Finland | Not stated | Randomized control trial | Hempseed oil | Oral | 20 | Atopic Dermatitis | (1) TEWL  (2) Plasma fatty acid level  (3) Rating scale of atopic symptoms including skin dryness, skin itchiness and use of medication |

|  | **Country** | **Cannabis-Related properties** | **Study Design** | **Interventions** | **Route** | **Sample**  **Size** | **Conditions** | **Outcomes** |
| --- | --- | --- | --- | --- | --- | --- | --- | --- |
| **Yuan et al.** | China | PEA/AEA | Randomized control trial | Emollient PEA/AEA cream | Topical | 60 | Asteatotic eczema | (1) EASI  (2) Skin surface hydration  (3) TEWL  (4) Perception threshold |
| **Rao A. et al.** | Australia | PEA formulation (Levagen+) | Randomized control trial | A moisturising base cream with 1.5% PEA (Levagen+) | Topical | 65 | Atopic dermatitis | (1)Self-Assessed Eczema Area and Severity Index (SA-EASI)  (2)pruritus Numerical Rating Scale (NRS)  (3)Patient-Oriented Eczema Measure (POEM)  (4)Dermatology Quality of Life Index (DLQI) |
| **Grant et al.** | United States of America | Dronabinol | Randomized control trial | 5–15 mg/day of dronabinol | Oral | 16 | Skin picking disorder | (1) The National Institute of Mental Health (NIMH) Symptom Severity Scale  (2) Self-report Massachusetts General Hospital Hair Pulling Scale (MGH-HPS) or the self-report MGH-HPS version for Skin Picking (3) The Sheehan Disability Scale |
| **Oláh et al.** | Hungary  Germany | Alkylamides  Alkylamides | Randomized control trial | Echinacea purpurea extract  Echinacea purpura extract | Topical  Topical | 60  25 | Atopic Dermatitis  Atopic Dermatitis | (1) SCORAD  (2) TEM analysis (3) Lipid analysis |
| **Ali et al.**  **(2015)** | Pakistan | Not stated | Quasi-experimental | 3% Cannabis sativa extract | Topical | 11 | Healthy Population | (1) Sebum level  (2) Erythema level  (3) Patient questionnaire including irritant, erythema and seven parameters of skin care properties |
| **Vitek et al.** | Slovenia | Not stated | Quasi-experimental | hempseed or flaxseed oil-based lyotropic liquid crystals | Topical | 12 | Healthy subject | 1. TEWL 2. SC hydration 3. Erythema index 4. Melanin index 5. Tolerability |
| **Ali et al.**  **(2020)** | Pakistan | Not stated | Quasi-experimental | Cannabis sativa extract | Topical | 11 | Healthy Population | (1) Texture parameter (energy, contrast, and variance)  (2) SELS (SEr, SEsc, SEsm, SEw) |
| **Rukwied**  **et al.** | United Kingdom | Cannabinoid receptor agonist  HU-210 | Randomized control trial | HU-210 Skin Patch | Topical | 20 | Healthy Volunteer with Capsaicin induced pain and hyperalgesia | (1) Pressure sensitivity  (2) Heat pain threshold  (3) Pinprick hyperalgesia  (4) Touch allodynia |
| **Gao et al.** | China | CBD | Randomized control trial | Pure CBD from hemp extract | Topical | 57 | Atopic dermatitis patients | ISGA scale |
| **Palmier et al.** | Italy | CBD | Observational study | Hemptouch skin ointment | Topical | 20 | Atopic Dermatitis,  Psoriasis,  Resulting scar | (1) TEWL  (2) Elasticity  (3) Hydration level  (4) SCORAD score  (5) PASI score  (6) ADI score |

| **Spiera et al.** | North America | Lenabasum | Randomized control trial | Lenabasum 20 mg twice daily, Lenabasum 5 mg twice daily | Oral | 365 | Diffuse cutaneous systemic sclerosis (dcSSc). | 1. The American College of Rheumatology combined response index in dcSSc (CRISS) 2. The modified Rodnan skin thickness score (MRSS) 3. physician global assessment of health 4. patient global assessment of health |
| --- | --- | --- | --- | --- | --- | --- | --- | --- |
| **Werth VP et al.** | United States of America | Lenabasum | Randomized control trial | 20 mg daily for 28 days, then 20 mg twice daily for 56 days | Oral | 22 | Dermatomyositis | 1. Cutaneous Dermatomyositis Disease Area and Severity Index (CDASI) activity 2. Patient VAS including pain and itch score 3. Skindex-29+3 4. PROMIS-29 |

**Supplementary table 1.** Characteristic of included studies

TEWL Transepidermal water loss, SCORAD Scoring Atopic Dermatitis, ADI Acne disability Index, PASI Psoriasis Area Severity Index, PEA N-Palmitoylethanolami, VAS Visual Analog scale, VRS Verbal rating scale, AEA N-arachidonoylethanolamide, EASI Eczema Area and Severity Index, TEM Transmission Electron Microscopy, SELS surface evaluation of the living skin, SEr skin roughness, SEsc skin scaliness, SEsm skin smoothness, SEw skin wrinkles, ISGA Investigator’s Static Global Assessment
