# Supplementary material for: Cannabis and cannabinoids in dermatology: a systematic review and meta-analysis of quantitative outcomes
Source: Front Pharmacol. 2025 Oct 17;16:1609667. doi: 10.3389/fphar.2025.1609667 (PMC12575346; doi:10.3389/fphar.2025.1609667)
Supplement: Supplementary file 6 [file Supplementaryfile1.zip › Supplementary table 2.docx]

**Supplementary Table 2** Intervention Characteristics of Meta-Analyzed Studies

| Study | Intervention | | | |
| --- | --- | --- | --- | --- |
|  | Types | Dose | Formulation | Duration |
| Ali et al.  (2015) | 3% Cannabis seeds extract cream | 2% ABIL EM 90, 14% Paraffin oil, 3% Cannabis sativa seeds aqueous methanolic extract. 1% fragrance (The plant material, crushed (40g) was extracted with solvent-aqueous methanol) | Cream | 12 weeks |
| Callaway et al.  (2005) | Hempseed oil | Cold-pressed from hempseed’s that was cultivated in Finland during 2001 then were bottled without any addi- tives, in unlabelled 200-ml brown glass and stored at +5 ̊C until use. | Oil | 8 weeks |
| Dvorak et al.  (2003) | 8 mmFinn chambers containing HU-120 | 50 ml of 50 mM HU210 | Skin patches | 24 hours |
| Gao et al.  (2022) | pure CBD from hemp | Not stated | Topical formulation | 14 days |
| Olah et al.  (2017) | Echinacea purpurea extract | CO2-extraction of Echinacea purpurea roots, Water-in-oil emulsion type and consists of the following ingredients: Aqua, Decyl Oleate, Isopropyl Myristate, Carthamus Tinctorius Seed Oil, Hexyldecanol, Hexyldecyl Laurate, Glycerin, Polyglyceryl-3 Poly- ricinoleate, Sorbitan Isostearate, Cera Alba, Citrus Aurantium Dulcis Peel Extract, Zinc Stearate, Benzyl Alcohol, Echinacea Purpurea Root Extract, Magnesium Sulfate, Lecithin, Tocopherol, Ascorbyl Palmitate, Glyceryl Oleate, Glyceryl Stearate, Citric Acid. | Cream | 3 months |
| Rao et al.  (2023) | moisturising base cream with PEA (Levagen+) | 1.5% PEA  The moisturising base cream was a water-based white viscous cream containing water, Camellia oleifera oil, mango seed butter, Jojoba oil, almond oil, calendula oil, panthenol, tocopherol, and a blend of essential oils for fragrance (lavender, geranium, and vanilla | Cream | 4 weeks |
| Visse et al.  (2017) | Physiogel® Calming Relief A. I. Body Lotion (containing PEA (S1); “PEA lotion” | Not stated | Lotions | 2 weeks |
| Werth et al.  (2022) | Lenabasum | 20 mg lenabasum daily for 28 days and then 20 mg twice per day for 56 days | Oral | 8 weeks |
| Yuan et al.  (2014) | Emollient PEA/AEA cream | purified water, Olea europaea, glycerol, pentylene glycol, palm glycerides, olus, hydrogenated lecithin, squalane, 0.3% PEA, 0.21% AEA, acetamide, monoethanolamine, betaine, sarcosine, hydroxy- ethylcellulose, sodium carbomer, carbomer, and xanthan gum | Cream | 4 weeks |
